# Complex Analyses of Plankton Structure and Function

**DOI:** 10.1100/tsw.2001.19

**Published:** 2001-04-04

**Authors:** Karl E. Havens

**Affiliations:** South Florida Water Management District, West Palm Beach, Florida 33416-4680, USA

**Keywords:** plankton, methods, size structure, carbon transfer, ecological transfer efficiency, algal-zooplantkon interactions, grazing, pelagic communities

## Abstract

This paper critically evaluates some complex methods that have been used to characterize the structure and function of freshwater plankton communities. The focus is on methods related to plankton size structure and carbon transfer. The specific methods reviewed are 1) size spectrum analysis, 2) size-fractionated phytoplankton productivity, 3) size-fractionated zooplankton grazing, 4) plankton ecological transfer efficiency, and 5) grazer effects on phytoplankton community structure. Taken together, these methods can provide information on community ecological properties that are directly related to practical issues including water quality and fisheries productivity. However, caution is warranted since application without a complete understanding of assumptions and context of the manipulations could lead to erroneous conclusions. As an example, experimental studies involving the addition or removal of zooplankton, especially when coupled with nutrient addition treatments, could provide information on the degree of consumer vs. resource control of phytoplankton. Resource managers subsequently could use this information in developing effective measures for controlling nuisance algal biomass. However, the experiments must be done critically and with sufficient safeguards and other measurements to ensure that treatments (e.g., zooplankton exclosure by screening of water) actually are successful and do not introduce other changes in the community (e.g., removal of large algae). In all of the methods described here, the investigator must take care when generalizing results and, in particular, carry out a sufficient number of replications to encompass both the major seasonal and spatial variation that occurs in the ecosystem.

## INTRODUCTION

The interactions between zooplankton consumers and their phytoplankton prey can have significant effects on pelagic ecosystem dynamics. Under certain conditions, zooplankton can suppress phytoplankton biomass, resulting in distinct clear water phases[1,2], while at other times zooplankton have little or no control over the phytoplankton, so that if nutrient inputs are high, dense algal blooms occur[3]. These blooms can be ecologically harmful, impair drinking water quality, and render a lake unsuitable for swimming and other recreational uses. Hence the phytoplankton-zooplankton interaction can be directly related to lake water quality as it pertains to important uses by society. The extent to which phytoplankton primary productivity is transferred to large zooplankton also can determine the productivity of commercial and sport fish, many of which depend on zooplankton as a primary food resource at some stage in their life[4,5,6]. In addition, the rapid growth rate and small size of plankton have made it an excellent research model for studying natural community dynamics as well as community responses to anthropogenic stress. There have been hundreds of papers published dealing with trophic interactions in the lake plankton. In the course of that research a number of novel methods have arisen for addressing complex properties, which I define here as properties at a higher order than standard measures of abundance, biomass, or productivity. A complex analysis, for example, might consider phytoplankton productivity, but also determine how the assimilated carbon is transferred to different size classes of zooplankton.

My objective is to review some of the methods that have been used to quantify complex properties of the freshwater plankton. These methods are important because they quantify "emergent" system-level properties that cannot be predicted by the "sum of the parts" or species by species manipulations. However, with any analytical method, particularly those involving size-selective manipulation of natural plant and animal communities, there are strengths and weaknesses. Accordingly, this review is a critical one, pointing out where investigators must use caution in their interpretation of results. The specific methods reviewed are not all-inclusive, but rather focus on five particular analytical approaches: 1) size spectrum analysis, 2) size-fractionated phytoplankton productivity, 3) size-fractionated zooplankton grazing, 4) plankton ecological transfer efficiency, and 5) grazer effects on phytoplankton community structure.

## SIZE SPECTRUM ANALYSIS

Pelagic food webs are comprised of series of progressively increasing body sizes[7], a property that is not found in most terrestrial communities[8]. In pelagic food webs the regularity in structure has facilitated the development of simple models of trophic interactions[9,10,11] and quantitative size-based predictions of fisheries production[12,13]. The models and predictions generally are based on the approach described by Sheldon et al.[7], wherein one constructs a two-dimensional graph showing the population biomass of progressively larger taxa versus the equivalent spherical diameter (ESD) of individuals in those populations. There are numerous variations to the approach; some researchers plot densities rather than biomass on the Y-axis, and some plot biomass or even biomass intervals on the X-axis. In the approach used here (Fig. 1), the ESD of each species was calculated by determining the diameter of a sphere having the same biomass as an average individual in the population under consideration, and the population biomass data were derived from microscopic counts and measurements. As is typically the case, data are displayed with the ESD values on a log-transformed axis, due to the wide size range that is encompassed by phytoplankton and zooplankton.

Another approach to presenting size spectra is to normalize the data[14,15] on the Y-axis by plotting the ratio of biomass divided by change in biomass across each size class in the community. For example, if a size class of phytoplankton included cells in the ESD range from 5 to 10 µm and the total biomass of that size class was 50 µg $l^{-1}$, then a "normalized size spectrum" would show that datum at 7.5 (the ESD mid-point) on the X-axis and 50/5 = 10 on the Y-axis. The features of interest in normalized spectra are 1) the downward slope of the data, and 2) the amount of scatter around a linear regression fitted to the data. The slopes tend to be considerably more negative for the open ocean than for inland lakes[15], indicating that a given amount of small algae in lakes results in a greater amount of zooplankton than it does in the ocean system. Size spectra for open ocean communities also tend to be relatively uniform, while those from freshwater lakes often have a greater scatter about the regression line, especially when those lakes are highly productive[15,16]. Some have concluded that this scatter represents departure from a steady state, and that it is evidence of a greater level of disturbance in lakes caused by factors including land-water interaction (nutrient runoff, allochthonous carbon inputs, pollutant loading, etc.)[17,18].

At the plant-animal interface in freshwater plankton, there is a clear relationship between the efficiency of carbon and energy transfer in the food web and the ESD difference of consumers and their prey[19]. Where small zooplankton and large phytoplankton co-exist, the ESD difference is small (Fig. 1A), and there tends to be low transfer efficiency. Under these conditions, which are characteristic of acid-stressed[20], metal-polluted[21], and highly eutrophic systems[22,23], the ratio of zooplankton to phytoplankton biomass also tends to be low. Ability of zooplankton to control the phytoplankton by grazing is minimal[24,25,26], and there generally is a reduced transfer efficiency of carbon to higher trophic levels[27,28]. In contrast, where phytoplankton ESD is small and zooplankton ESD large (Fig. 1B), there generally is a greater transfer efficiency.

In carrying out a size spectrum analysis, it is important to be aware of confounding effects of plankton taxonomic structure. For example, there might be a lower efficiency of transfer if the large zooplankton in Fig. 1B is cyclopoids, rather than cladocerans, because the most abundant phytoplankton (~2 µm ESD) is not directly available to those consumers[29]. Cyclopoid copepods, in addition to feeding on small zooplankton, consume only the largest net-phytoplankton, such as dinoflagellates[30]. Cladocerans, on the other hand, and in particular large *Daphnia* can graze a wide range of particles including small nano- and pico-plankton cells[31,32,33,34]. Hence, in a cyclopoid-dominated community, there may be multiple steps in the food web linking the phytoplankton to the zooplankton grazers (e.g., pico-plankton → heterotrophic protozoa → rotifers → cyclopoids). In contrast a *Daphnia*-dominated community might have primarily a two-step transfer (pico-plankton → *Daphnia*), less respiratory carbon loss, and greater transfer efficiency[32,33,35]. It is noteworthy that communities with strong *Daphnia* dominance appear to display distinctive size spectra, with accumulation of biomass in a small number of size classes (*Daphnia* and large inedible algae) and the occurrence of size classes (nanoflagellates and small ciliates) with little or no biomass[18].

Other factors can influence the relationship between size spectra and ecological interactions. These include predator deterrents, such as gelatinous sheaths, spines, and noxious chemicals[3,36].

In conclusion, size spectrum analysis can provide insight into the functionality of the community without a need to

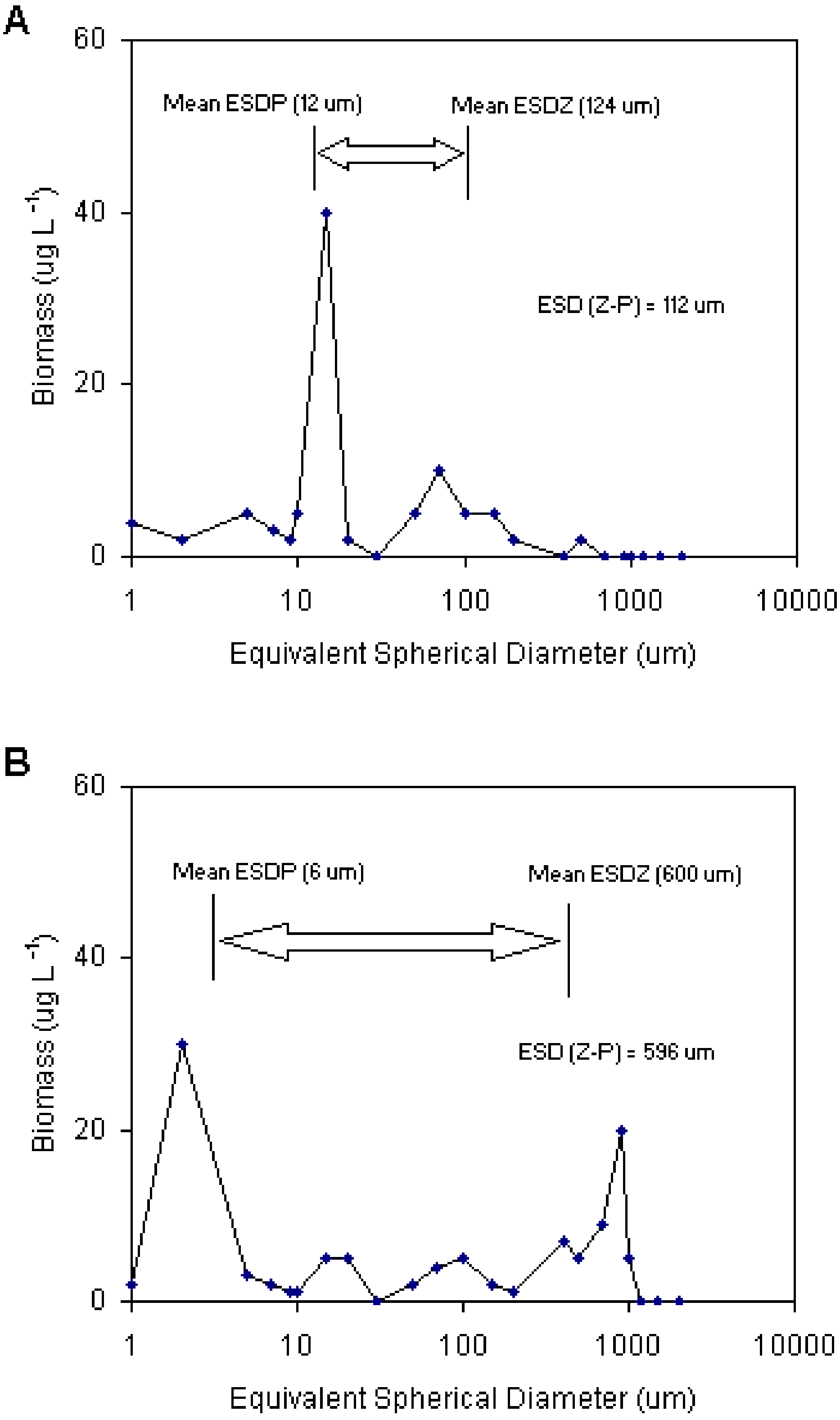

**FIGURE 1.** Size spectra[7] for two hypothetical plankton communities, plotting the biomass of each population as a function of the average size of its individual members, with size being expressed as the equivalent spherical diameter (ESD). Biomass-weighted mean ESD values also are provided for phytoplankton (ESDP) and zooplankton (ESDZ), and the size difference (ESDZ-P) is calculated for each example. (A) A community with large phytoplankton and small zooplankton. (B) A community with small phytoplankton and large zooplankton.

carry out more costly and labor intensive network analyses. However, caution must be used in interpreting the results because attributes other than size can sometimes influence the realized community functions. Analyses that use only size information (e.g., from a particle counter) are therefore discouraged.

## SIZE-FRACTIONATED PHYTOPLANKTON PRODUCTIVITY

Because the size of phytoplankton affects their availability to zooplankton grazers[37] and hence the extent to which photosynthetic carbon is transferred upward in the food web, many investigators have carried out assays to directly measure the contribution of various sized phytoplankton to primary productivity. Size categories most commonly considered are pico-plankton (0.2 to 2 μm), nano-plankton (2 to 20 μm), and micro-plankton (>20 μm), after Sieburth et al.[38]. These measurements, like size-structure, can lead to important practical applications. For example, in Canadian lakes where primary production was dominated by pico-plankton, the production of commercial fish (salmon) was low[39]. When nutrients were added to some of these lakes, the productivity of larger algae increased and the production of salmon increased by severalfold[40]. These results were consistent with models based on phytoplankton productivity studies, which served to guide the management actions.

Size-fractionated productivity can be measured using the standard $^{14}C$ light and dark bottle method of Vollenweider[41]. The only modification to the method is that the algae are size-fractionated either before or after the incubation period. There exist two opposing viewpoints regarding this aspect of the analysis. Proponents of a pre-incubation fractionation[42,43,44] argue that post-incubation filtration damages cells, causing a loss of radiolabel into the smaller size fractions. They suggest that this gives rise to an overestimate of the importance of pico-plankton productivity. Proponents of post-incubation fractionation argue that their approach minimizes stress to the cells before incubation, which presumably could cause unnatural rates of carbon uptake. Furnas[45], for example, argued that screening of cells before incubation results in trauma, especially to small pico- and nano-plankton cells, and also noted that screened samples might behave differently on incubation because of uncoupling of tight interactions between the different size classes of cells.

One might conclude that the two methods could give nearly identical results as long as care is taken in the filtration process to minimize damage to fragile cells. This issue has been recognized for some time[46,47] and remedies have been suggested. Filtration always should be done under a vacuum pressure to avoid the rupturing of cells and only small volumes of water should be filtered, to avoid "packing" of multiple layers of cells onto the filter. Nevertheless, Fahnenstiel et al.[44] concluded that the pre-incubation fractionation gave biased results (erroneously high pico-plankton productivity) in the Laurentian Great Lakes, even when steps were taken to avoid filtration artifacts. Likewise, Furnas[45] found a bias in the results from pre-incubation screening, despite "stringent efforts to carry out size separations gently." This suggests that if one intends to carry out a size-fractionated productivity study, some preliminary comparisons should be made between results using the two approaches. If significant differences are observed, additional investigation might be considered to identify the underlying cause. In Ohio and Florida, U.S. lakes where I have made this comparison, nearly identical results have been obtained[48]. I subsequently have used the post-incubation approach because it is less time consuming.

Analysis of size-fractionated productivity can be carried out in tandem with a quantitative analysis of size-fractionated biomass (based on microscopic counts or analysis of pigment or carbon content). I have used this tandem approach to study the plankton of a subtropical lake[48], and it recently was used to evaluate the phytoplankton of Tokyo Bay[49]. In these examples there was a close correspondence between the biomass and productivity data, indicating that biomass-specific production did not vary much with cell size. However, this is not always the case[50], and that information is important when interpreting the results of zooplankton grazing experiments (see below) that use the natural phytoplankton as a food source.

One major criticism of size-fractionated productivity studies is that they are based on operationally defined size categories of phytoplankton, which may or may not correspond to categories that are taxonomically distinct in the community. A given filter size may split a certain species between two size classes. I have encountered this problem when the dominant algal cells are elongated (e.g., *Syndera* and other pennate diatoms) with a length that is in excess of the mesh size and a width that is considerably smaller, and when colonial forms (e.g., *Chroococcus*) occurred. The filter then retains large colonies, but allows smaller colonies or individual cells of the same species to pass through. Furthermore, the often used 2–20 μm nano-plankton size class can sometimes contain a wide range of phytoplankton functional groups. These include naked flagellates that are readily grazed by small and large zooplankton, and larger diatoms and dinoflagellates that are available only to calanoids and larger cladocerans[51].

Depending on plankton community structure, certain filters also may be subject to clogging, producing strong filtration artifacts in studies of size-fractionated biomass or productivity. Carrick and Schelske[52], for example, found that pico-plankton accounted for approximately half of total phytoplankton biomass in a hypereutrophic Florida lake, and documented that this result would not have been found using standard filtration methods. Membrane filters with a 5.0-μm porosity became clogged with filaments of *Lyngbya* and colonies of *Microcystis*, such that small pico-plankton did not pass through. This problem was documented by carrying out microscopic counts of the community, and it occurred even when small volumes of lake-water (50 ml) were filtered. Pico-plankton biomass was underestimated by as much as 80%. The authors found that, in this particular case, the problem could be largely overcome by using large-capacity filter "capsules" (Whatman 5.0 μm Polycap®).

Hence, as with the size spectrum analysis, interpretation of results from size-fractionated productivity studies requires an investigator to carefully consider the full context of the taxonomic structure of the community. At a minimum, control studies should be performed in order to evaluate what actually is passing through vs. being retained by each successive filter, as well as the aforementioned comparisons of pre- vs. post-incubation fractionation procedures. In some cases, nontraditional methods of filtration may also be called for.

## SIZE-FRACTIONATED ZOOPLANKTON GRAZING

In a similar manner one can evaluate the grazing rates of different size classes of zooplankton. The results could be important in determining, for example, what amount of the primary productivity is grazed by animals that are large enough to be readily available to fish.

Three methods have been used, the most common being that generally referred to as the Haney[53] chamber method, although the experimental device is actually a modified version of the chamber designed by Gliwicz[54]. The chamber is a clear plastic cylinder with plate-like doors at either end that are closed when a messenger is dropped down a supporting line to the submersed device. When the doors close, zooplankton is captured inside the chamber, in the same manner that a water sample is captured inside a VanDorn bottle. At the same time that this occurs, a small vessel inside the chamber is triggered to open, releasing some food source (algae, bacteria, yeast, or plastic micro-spheres) that were held inside in solution. The food disperses through the chamber and the animals feed for some specified time period, after which the chamber is retrieved. Gliwicz[54] measured grazing rates by comparing the final density of food particles in chambers where zooplankton were either anaesthetized with a chemical (physostigminum salicylium) or left alone and allowed to graze naturally. Haney[53] modified the device and used radiolabeled food. This allowed for a more precise estimate of grazing rate because measurements were made directly on the zooplankton, rather than comparing food densities among different chambers. After some specified time has elapsed (less than the gut passage time of the dominant grazers[55]), the chamber is retrieved and the macro-zooplankton and micro-zooplankton are size-fractionated with successive screens (e.g., 200 and 40 μm). Filtering rate (F, ml $l^{-1}$ $h^{-1}$) is determined for each zooplankton size class based on the measured activity of the grazers and the activity of an aliquot of feeding suspension collected from the chamber after the incubation. The following formula is used:

$$F = (\text{dpm ml}^{-1}\ \text{grazers})/(\text{dpm l}^{-1}\ \text{feeding suspension}) * T \quad (1)$$

where dpm is disintegrations per minute measured with a liquid scintillation counter, and T is a temporal conversion factor (e.g., if the incubation period was 5 min, T would be 60/5 = 12). If information is available regarding the carbon biomass of phytoplankton (μgC $l^{-1}$), one can convert these results into units of carbon flux rate (μgC $l^{-1}$ $h^{-1}$) by multiplication.

The strength of the Haney[53] chamber method is that it allows zooplankton to graze under natural conditions, with no stress due to handling prior to incubation. The method can be modified using differently labeled algae ($^{14}C$) and bacteria ($^{3}H$) so that simultaneous measurements of F can be made for the two food types[56]. Alternatively, some investigators carry out separate runs of the experiment with radiolabeled bacteria and radiolabeled algae. It also is possible to use food items that are fluorescently labeled so that microscopic examination of zooplankton can show which grazer species consume the different food types[57]. Indeed a combined use of radiolabeled and fluorescently labeled foods representing a variety of naturally occurring taxa is an excellent way to elucidate both the structure and function of the plankton food web[58]. The only significant drawback to these methods is that one does not calculate F using the natural phytoplankton community as the food source. Rather, F is approximated based on experiments using a small number of "model" plankton taxa. Most studies in the literature have used small flagellates (e.g., *Chlamydomonas*, *Rhodomonas*, *Cryptomonas*) as algal models. I typically use at least three models — a small flagellate, a moderate-sized diatom, and a filamentous blue-green, but still this does not always encompass the variability in the natural community. It also is difficult to predict which models will be most appropriate for any particular date or location where an experiment occurs (see below).

An alternative approach for measuring grazing rates of micro- and macro-zooplankton was described by Bogdan and McNaught[59] and later modified for *in situ* use[60,61]. A large volume of lake water (e.g., 20 l), complete with its natural phytoplankton, is screened to remove zooplankton and then exposed to a high activity of radiotracer (e.g., $^{14}C$ bicarbonate at 10–50 μCi $l^{-1}$). Zooplankton is collected from the lake and placed into a cylindrical "grazing chamber" constructed from a section of clear plastic pipe (10-cm diameter) with a Nitex® screen on the lower end to retain the animals. Care is taken to pour water from the collection device into the partially submerged grazing chamber so that grazers experience a minimal amount of stress in the procedure. Once a suitable amount of zooplankton (from a known volume of water) has been collected, the tube is quickly raised from the water and immersed into the container of radiolabeled phytoplankton. At the end of a short incubation period, the tube is removed and the zooplankton is captured once again on the screen. The animals are rinsed from the screen into a vial with scintillation cocktail for determination of radioactivity. The activity of feeding suspension also is measured and Equation 1 is used to determine F.

I have used this approach with grazing chambers having two sizes of Nitex mesh — a 200-µm mesh chamber for measuring grazing by macro-zooplankton, and a 40-µm mesh for measuring grazing by the entire zooplankton community. The grazing of micro-zooplankton is determined by difference. The method also can allow for a determination of size-selective grazing by the two zooplankton groups (Fig. 2). This is accomplished by adding one step to the process described above, wherein the radiolabeled algae is size-fractionated after its incubation with a 20-µm mesh, and then reconstituted with unlabeled size-fractionated algae. This produces two complete feeding suspensions for use in the grazing study, one with labeled nano-plankton and one with labeled micro-plankton.

This method has some advantages over the Haney[53] chamber approach: 1) it allows the investigator to use natural phytoplankton as the food source in the incubations, and 2) it allows larger volumes of water to be collected for obtaining zooplankton. Haney chambers typically are 2 to 8 l in size. In my experience, a 4-l chamber is the maximal size that can be easily lifted in and out of a small boat. Except in eutrophic lakes, 4 l of water may not contain a sufficient density of macro-zooplankton to allow accurate measurement of F (due to low radioactivity relative to background). With the alternative approach, one can filter large volumes of water to collect zooplankton, thereby avoiding this problem. The disadvantage is that this collection procedure might stress the zooplankton prior to incubation, and perhaps affect their grazing rates.

It is prudent to carefully weigh the pros and cons of the two methods in the context of the questions being asked. Preliminary comparative studies are particularly valuable, if both pieces of equipment are available. In Lake Okeechobee, Florida, I found that with careful handling of animals, zooplankton community F estimated from the Haney[53] method was within 10–20% of that determined with the method of Bogdan and McNaught[59]. Furthermore, the results suggested that the Haney[52] method (which used cultured natural bacteria as a model for pico-plankton, *Chlamydomonas* as a model for nano-plankton, and *Anabaena* as a model for micro-plankton) underestimated F for micro-plankton[48]. This is because the natural community was dominated by smaller taxa of micro-plankton (*Merismopedia* and *Aphanocapsa*) that were more readily filtered from the water by grazers. Our choice of *Anabaena* as a model was based on data regarding typical community structure for that time of year, but on the sampling day, this did not agree with what actually was found. Unless one uses a large variety of algae models and then selects the results from those that match the natural community (this would be very tedious and inefficient), this situation is not unexpected when using the Haney method.

As indicated above, the end result of the grazing study is an estimate of community filtering rate, F, which can be multiplied by some estimate of algal or bacterial carbon biomass to determine carbon flux. An important consideration at this stage is determining which fraction of the algal assemblage actually is grazed, so that an appropriate multiplier is used. For example, in a phytoplankton community comprised of 60% filamentous cyanobacteria, 20% pennate diatoms, and 20% nano-flagellates, the zooplankton might only consume the latter group. As such, the biomass of nano-flagellates, rather than total phytoplankton biomass, should be the multiplier in calculation of carbon flux rate. Addressing this issue requires a careful microscopic analysis of the gut contents of dominant grazers. As indicated above, grazing studies with fluorescent bacteria can be used to identify zooplankton that feed on these cells[57], which are too small to observe in light microscope analysis of gut contents.

Although it has not been widely used, there is a third method for measuring *in situ* grazing of small vs. large zooplankton. Mazumder et al.[62] constructed a device that has features in common with the Haney chamber. Three 4-l grazing cylinders are mounted side-by-side on a frame, so that when immersed and triggered, zooplankton grazing studies can be done simultaneously with injection of three different kinds of radiolabeled or fluorescent food particles (alternatively, three replicates of the same experiment could be performed). Coupled with the grazing chambers is a size-fractionating device that allows immediate screening of the zooplankton into the categories described above. Rather than using cultured algal models, the authors used the radiolabeled natural phytoplankton as the food source in the experiments. They carried out studies where particular size fractions of natural phytoplankton (0.2–1, 1–3, 3–8, and 3–20 µm) were used to estimate F. With this device, up to 186 separate estimates of zooplankton community filtration were obtained during an 8-h sampling period. The complex design and relatively large size of the grazing and filtration apparatus (Fig. 1 in Mazumder et al.[62]) may be a reason that this custom-made device has not gained more widespread use among plankton ecologists.

Clearly each of these methods has its advantages and disadvantages, related to ease of use, maintenance of near-

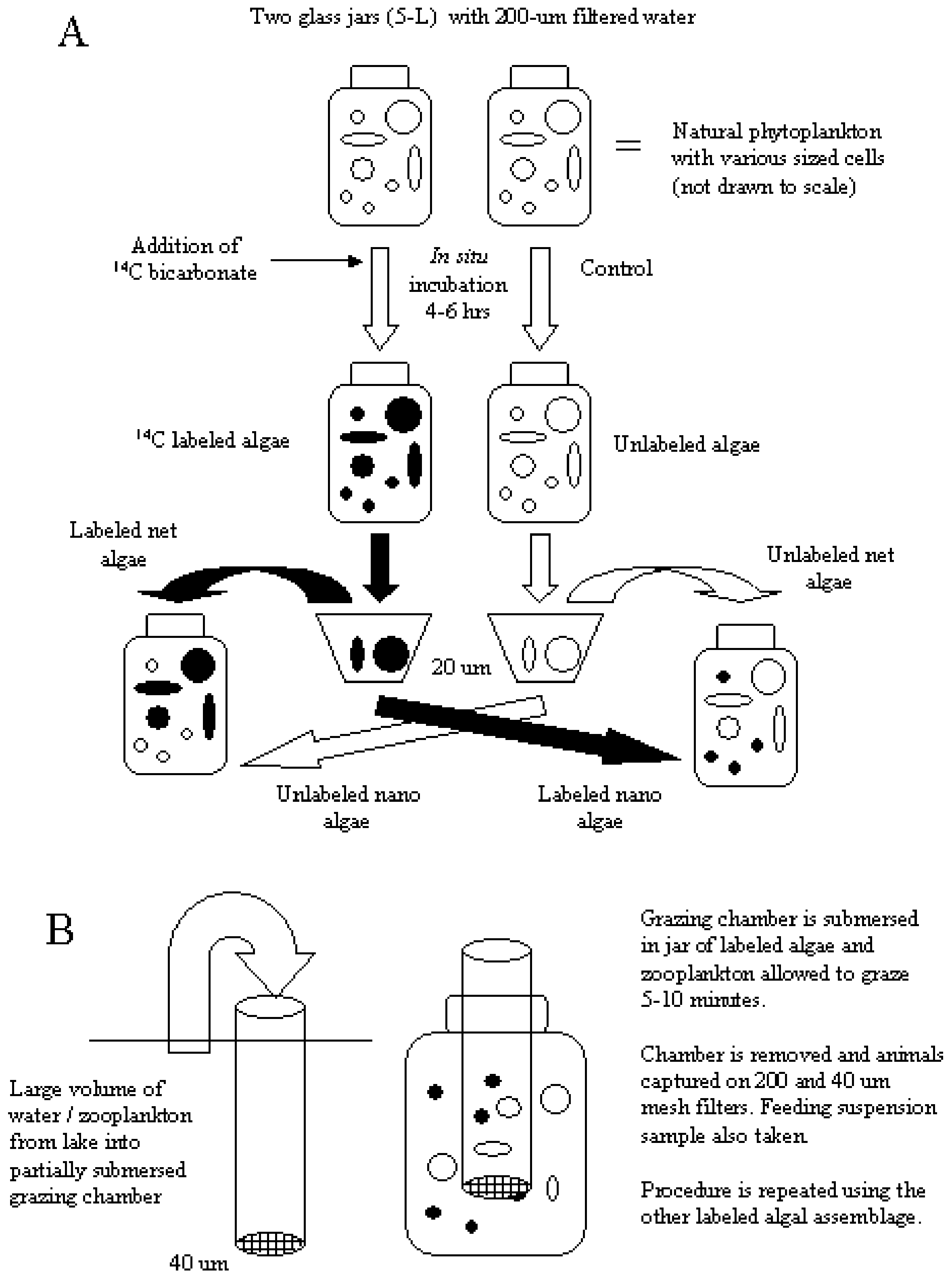

**FIGURE 2.** The design of a natural community grazing experiment[61] whereby one can determine the grazing rates of macro- (>200 mm) and micro-zooplankton (40-200 mm) on both nano- (<20 mm) and micro-phytoplankton (>20 mm). (A) The process used to establish two complete algal communities with different size fractions labeled with $^{14}C$. (B) The process used to perform the grazing experiments.

natural conditions, and ability to use natural vs. lab-grown food items. Depending on the research questions that are posed and the weight that is placed on these different factors, any one of the methods might represent the optimal choice. The key, as with the other complex analyses described here, is for the investigator to have a sound understanding of the assumptions that are inherent in using any particular approach. This will minimize the chance of erroneous conclusions.

## ECOLOGICAL TRANSFER EFFICIENCY

The extent to which the carbon fixed by phytoplankton is transferred upward in the food web to macro-zooplankton can largely determine the productivity of fish in pelagic systems[39,63]. The efficiency of this carbon transfer can be estimated from the ratio of zooplankton carbon uptake to phytoplankton carbon uptake, if the components are measured at comparable temporal and spatial scales. There are two general approaches for measuring transfer efficiency.

The first approach is to measure carbon flux rates to various size classes of phytoplankton and zooplankton, as described above, and then calculate the efficiency using the resulting data. Where this is done, it is important to recognize that a large portion of the carbon flux may occur in pathways with a basis in bacterial, rather than algal, production[32,34,64]. These microbial pathways (bacteria → protozoa → zooplankton) should be measured, along with the more traditional "grazing chain" (algae → zooplankton), in calculating the overall efficiency of the web. Because of the complexity of the measurements required to fully assess carbon flow in plankton food webs, that particular approach has been carried out only for a limited number of ecosystems[58,65,66,67].

The second approach[68] is to measure carbon transfer by adding a radiotracer to the community and examining the transfer of activity over time into the macro-zooplankton size class. Koshikawa[64] used this method to compare efficiencies of bacterial and algal-based pathways of an inland sea in Japan, by carrying out the assays of macro-zooplankton in separate experiments where label was added as $^{13}$C-bicarbonate or $^{13}$C-glucose. Rather than using the term ecological transfer efficiency, they defined the attribute measured as percent label transfer (PLT), calculated as:

$$\text{PLT}\ (\%) = (^{13}C_{ex\text{-}zoop} / ^{13}C_{ex\text{-}all}) \times 100 \qquad (2)$$

where $^{13}C_{ex}$ of zooplankton captured on a 100-µm mesh (zoop) and of the complete plankton community ($^{13}C_{ex\text{-}all}$) are measured based on stable isotopic composition as:

$$^{13}C_{ex}\ (\mu g\ ^{13}C\ l^{-1}) = (a_s - a_n) \times \text{POC} \qquad (3)$$

where $a_s$ and $a_n$ are the $^{13}$C atom % in an incubated size fraction and a natural one, respectively, and POC is the particulate organic carbon measured (mg C $l^{-1}$) in that fraction. I recently used this method[69] to measure PLT from bacteria and algae to macro-zooplankton in a subtropical lake, but used radioisotopes rather than stable isotopes in the procedure. Natural bacteria cultured from the lake were labeled with $^{14}$C-glucose and algae were labeled with $^{14}$C-bicarbonate. In this case the PLT was calculated as the ratio of activity in the 200 µm size fraction (which better represented macro-zooplankton in the ecosystem) to total activity of particles greater than 0.2 µm. Separate experiments were done for calculating PLT associated with algae vs. bacteria.

Regardless of whether one uses stable or radioactive isotopes as tracers, this is a simple method for quickly measuring transfer efficiency in the plankton. However, there are a number of issues that must be critically considered in interpreting the results. First, the method makes use of size-fractionation. I generally filter water through the following sequence: 200, 40, 20, 2, and 0.2 µm (lesser volumes at each step), and then calculate the summed activity per unit volume of all fractions to get $C_{ex\text{-}all}$ in Equation 2. The activity of the 200-µm fraction gives the number analogous to $C_{ex\text{-}zoo}$.

If one considers a certain size class (e.g., 200 µm) to represent macro-zooplankton, then microscopic analysis of material actually retained and passed by the mesh should be carried out to document that this is indeed the case. In some lakes with seasonal variability in zooplankton size, one might consider adjusting the mesh size used to collect the macro-zooplankton fraction. Alternatively, the researcher might use only the operationally defined name of the size class, rather than attributing it to a particular type of plankton. It also is important to recognize that once a tracer is added to the community, there are various complex pathways whereby the C might ultimately reach zooplankton. Some of the carbon added as glucose and first taken up by bacteria might be respired and subsequently taken up by phytoplankton as radiolabeled bicarbonate. Likewise, it has been well established that much of the carbon taken up by phytoplankton is subsequently excreted as dissolved organic carbon, which then can be incorporated by bacteria into the microbial food web[70,71,72]. Hence the measurement of PLT by the methods described here does allow one to differentiate between efficiencies of carbon transfer originating at phytoplankton vs. bacteria, but not the efficiencies of grazing vs. microbial pathways *per se*. To accomplish the latter, one must carry out the more detailed analysis of food web structure and function described above.

Percent label transfer is likely to be a time-sensitive attribute, increasing to some maximal value after tracer addition after several hours and then declining to zero. Therefore comparisons between studies carried out at different times of the year or on different lakes should be done only when the incubation time is the same. Ideally one would evaluate the time course of PLT in the systems under comparison and contrast PLT maximum from each study.

## GRAZING IMPACTS ON PHYTOPLANKTON

Porter[51] carried out some of the earliest controlled experiments to evaluate effects of zooplankton grazers on natural phytoplankton, and documented that taxa regularly found inside the gut of grazers (e.g., small naked flagellates) displayed an increase in their biomass in treatments where grazers were removed. Porter[51] and others who subsequently performed such experiments recognized that when dealing with the whole plankton community, large volume experimental enclosures are required and *in situ* incubations are optimal. Porter[51] used transparent plastic bags (500 l) suspended underwater with surface floats and anchors as the experimental units. Other investigators have used large plastic cylinders (with open or closed bottoms) exposed at their surface to the atmosphere, and some have used plastic carboys (5 to 20 l) for short-term incubations. One noteworthy aspect of whole-community plankton experiments is that they generally can be performed at low cost using readily available materials. I have performed studies using clear plastic trash bags purchased at a local hardware store, nylon line, plastic milk jugs filled with foam, and concrete blocks (total cost below US $20 for the study).

Bergquist and Carpenter[73] expanded on Porter's original design by looking at phytoplankton responses in treatments spanning a wide range of zooplankton densities. They removed the natural zooplankton community by passing the water through a 75-µm screen, and then created treatments (by adding back the collected zooplankton) that had grazer densities ranging from 0.5 to 9X ambient. This study documented a unimodel relationship between phytoplankton growth response and zooplankton biomass. At the highest biomass of grazers, the phytoplankton was limited by intense predation. At the lowest biomass of grazers, the phytoplankton was likely limited by nutrients. At the intermediate grazer density, the balance of predation and nutrient regeneration by zooplankton was concluded to have a net positive effect on the phytoplankton growth. This was the first study to document the unimodal relationship between producer growth and grazer biomass, which had been observed earlier for certain terrestrial and attached algal communities. In a subsequent study, the same relationship was documented in survey data collected over a summer period in three experimental lakes (Paul, Peter, and Tuesday Lakes, Michigan)[74].

Lehman and Sandgren[75] carried out experiments that included both nutrient-addition and grazer removal treatments, in order to gain further insight into the relative importance of resources vs. consumers in controlling phytoplankton community structure. They found that species of phytoplankton that increased when grazers were experimentally removed (by filtration with a 130-µm mesh) were generally small naked flagellates, confirming what Porter[51] had found. However, other species declined when grazers were removed, suggesting that they may have benefited indirectly from the consumers' presence. Typically this phytoplankton was found to be nutrient-limited, i.e., the biomass was increased in the treatment where Lehman and Sandgren[75] added nutrients. This suggests that phytoplankton were experiencing a net gain in the amount of available nutrients due to the grazers, perhaps because the grazers were excreting readily available nutrients into the water. An alternative explanation, that the phytoplankton was passing unharmed through the guts of zooplankton and sequestering nutrients in the process was not supported by the results, although this phenomenon is known to occur[76].

Elser and Goldman[77] modified the design of these experiments one step further, and obtained estimates of both the direct and indirect effects of zooplankton on the phytoplankton. An experiment was designed under the premise that nutrient-saturated phytoplankton will be unaffected by further additions of nutrients that are contributed by zooplankton grazers. Therefore the grazing rate (independent of stimulation by nutrient release) on any phytoplankton taxon can be estimated by the difference in its net growth rate under nutrient-saturated conditions in the presence vs. absence of grazers. The experimental design included a factorial manipulation of nutrients and zooplankton. Treatments were controls (C, no nutrients, natural grazer densities), zooplankton removal (Z, removed with a 125-µm mesh), nutrient addition (N, both nitrogen and phosphorus added at saturating concentrations), and zooplankton removal plus nutrient addition (ZN). This design (Fig. 3) allows one to estimate the net effects of zooplankton (positive and negative) on phytoplankton, by comparing net phytoplankton growth in the C and Z treatments, and the grazing (negative) impact of zooplankton by comparing phytoplankton growth in the N and ZN treatments. The nutrient recycling effects (positive) of zooplankton on the phytoplankton then can be calculated by difference.

The method of Elser and Goldman[77] has been widely used, most recently in experiments at Lake Biwa, a large lake ecosystem in Japan[78]. In that study it was documented that during the summer phosphorus-limited period, epilimnetic bacteria-plankton were able to compensate for 30 to 40% of their grazing loss by uptake of nutrients released by the zooplankton. In deeper water, however, bacterial growth was limited by low water temperature and there were no positive effects of zooplankton observed (only grazing losses). These results varied with season; during winter when nutrient availability was greater, epilimnetic bacteria also did not benefit from zooplankton nutrient recycling. The findings are noteworthy because they demonstrate seasonal and spatial (in this case, vertical) variation in community responses to the treatments.

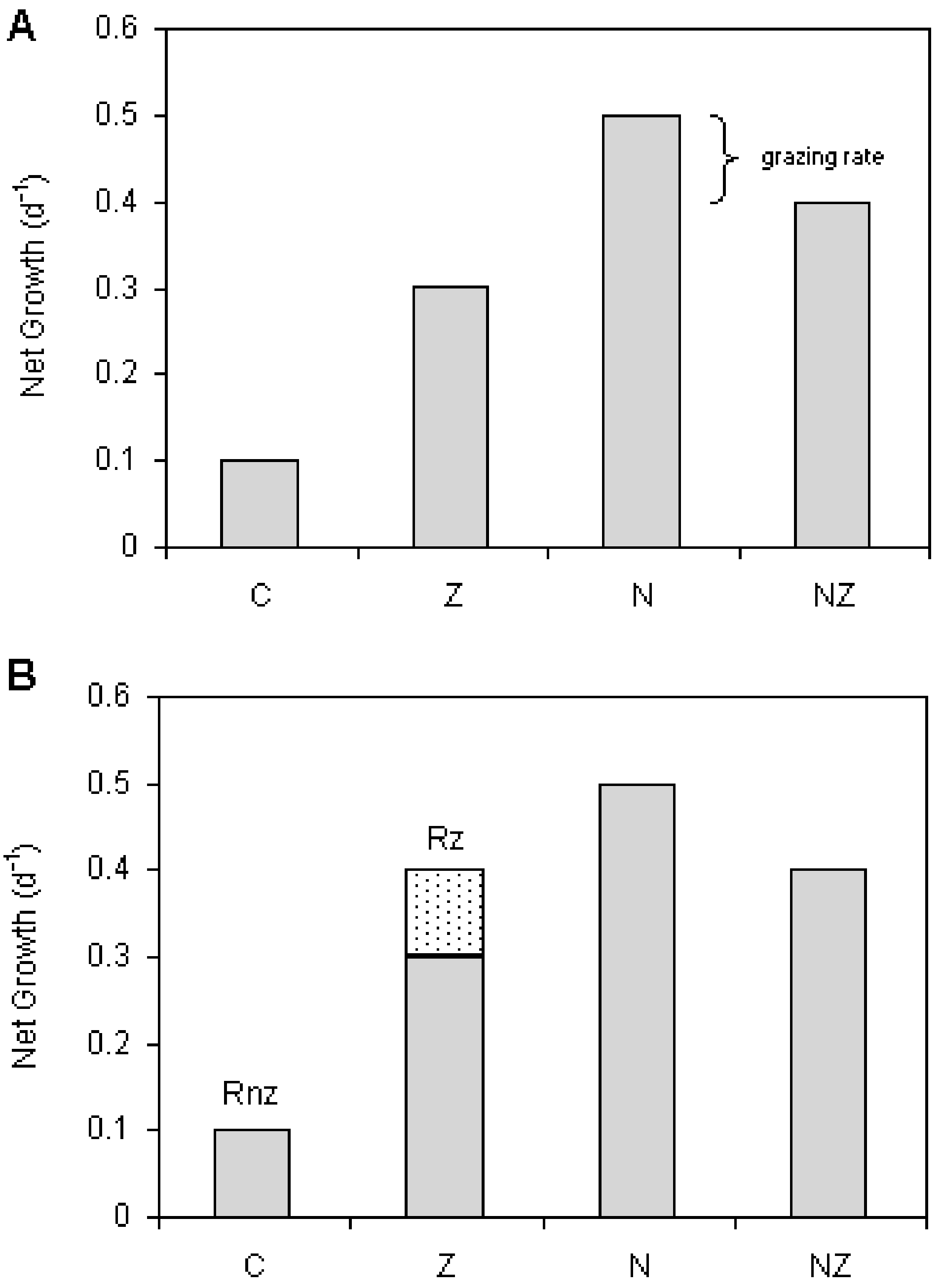

**FIGURE 3.** Interpretation of results from a controlled zooplankton/nutrient addition experiment.[77] Treatments include a control (C, zooplankton removed and no nutrients added), zooplankton addition (Z, zooplankton added at ambient densities, no nutrients added), nutrient addition (N, zooplankton removed and nutrients added at a high concentration), and nutrient + zooplankton (NZ, nutrients and zooplankton added as above). (A) For each algal species, the specific grazing rate can be calculated as the difference in growth in the N and NZ treatments. (B) The specific growth rate in the presence of zooplankton (Rz) can be calculated by adding the specific grazing rate to the observed growth rate in the Z treatment. This can be compared with growth in the absence of zooplankton (Rnz).

There have been a number of other variations on the basic experimental approaches described above, each providing additional insight into the functioning of the complex freshwater plankton community. For example, Pace and Funke[79] considered the responses to microbial components, as opposed to phytoplankton, to grazer and nutrient manipulations. They found that protozoa were significantly reduced when large Daphnia were added to enclosures, but bacteria-plankton were not affected. Similar results were obtained by Brett et al.[80], who carried out a complex study that investigated the effects of particular species of zooplankton on natural phytoplankton communities. In that case the lake water was filtered with an 83-µm screen, and then treatments were established by adding back high densities of *Daphnia*, *Holopedium*, *Diaptomus*, or *Diacyclops*. Where cladocerans or calanoids were added, there were significant reductions in the biomass of phytoplankton (in particular small species of *Peridinium* and *Rhodomonas*) and ciliates, but no effects on bacteria. Where cyclopoids were added, there were significant reductions in the biomass of rotifers and ciliates, but no effects on other plankton components. This study demonstrated in an elegant manner the functional role of different grazers in the community.

In general, studies of this nature have provided insight into the functioning of natural plankton communities, but as with the other analyses reviewed above, there are critical aspects that need to be considered. First, one cannot simply assume that a screening procedure results in "zooplankton-free" conditions. Preliminary studies should be performed to identify a mesh size that most effectively removes zooplankton grazers from the community and the contents of the enclosures should be filtered with a finer mesh at the end of the experiment and counted microscopically to determine actual grazer densities. In some cases where the zooplankton includes a high percentage of taxa with narrow bodies (e.g., *Tropocyclops*), a large percentage of the animals might pass the mesh. With reduced competition for food, their growth rates might be markedly increased, such that "zooplankton-free" treatments actually end up with relatively high densities, depending on the duration of the experiment[26]. To minimize the problem, the duration of zooplankton exclosure studies generally is held to 48 or 96 h, which is still a sufficient amount of time to observe significant phytoplankton community responses to both nutrients and grazer manipulations[73,74,75].

Another consideration in zooplankton exclosure experiments is the selection of a mesh size that does not remove a significant fraction of the dominant phytoplankton. In eutrophic lakes where zooplankton body size is small and phytoplankton are large, exclosure experiments are most challenging, because it is nearly impossible to remove macrozooplankton effectively without severely impacting the phytoplankton.

A final consideration that applies to any enclosure study is how the experimental device (bag, tube, limnocorral, etc.) changes the physics of the environment. Enclosing portions of a water column inside a rigid or semi-rigid device may result in rapid and differential setting of plankton[81], and in nutrient-deficient environments, the growth and subsequent nutrient uptake by periphyton on enclosure walls[82] might become a significant factor in controlling plankton responses. This topic is widely addressed in the literature, where investigators have used a variety of approaches to minimize the enclosure effects, including increased enclosure size (less wall surface per unit enclosed volume), shorter experiment duration, and in some cases, artificial mixing of the enclosed water.

Where the experiments are done carefully, taking into consideration the above- mentioned assumptions and limitations, they can be very effective in testing alternative hypotheses regarding factors controlling the phytoplankton. The results can be used to effectively predict whole-ecosystem responses to experimental manipulations[83], and they can lead to management recommendations such as fish manipulation or nutrient load reduction as means of reducing biomass of phytoplankton[84].

## SUMMARY

There are a number of complex yet powerful methods for quantifying the structure and function of plankton communities. Taken together these methods can provide information related to food web connectedness, energy flow, and interaction strength, in the sense that Paine[85] described these terms. While one can debate which type of information about the community is "best," it is clear that each approach provides unique and important information. Although not typically done, one might consider making use of the full array of the methods described here, in order to obtain the most complete understanding possible of the community under investigation. A critical point, regardless of the methods used, is that the work be carried out in a critical manner, and by an investigator with a solid understanding of the strengths and weaknesses of the approaches used. For example, almost any investigator could carry out the relatively simple and low-cost zooplankton exclosure experiment. However, to do these studies without adequate safeguards and other measurements risks conclusions that are incorrect, or worse yet, misleading to those who use the results to make decisions about how to manage the water resource. When done critically, however, the methods described here can provide rigorous scientific information. Under ideal circumstances, the short-term experimental methods should be combined with a long-term program to monitor changes in community structure and environmental conditions. Taken together, this would offer the most powerful approach for obtaining the information guiding resource management decisions.

## ACKNOWLEDGMENTS

This manuscript was substantially improved as a result of constructive comments provided by Hunter Carrick, Peter Doering, Therese East, Garth Redfield, and Alan Steinman.

| | | |
|---|---|---|
| Received: | February | 14, 2001 |
| Accepted: | March | 1, 2001 |
| Published: | April | 4, 2001 |
